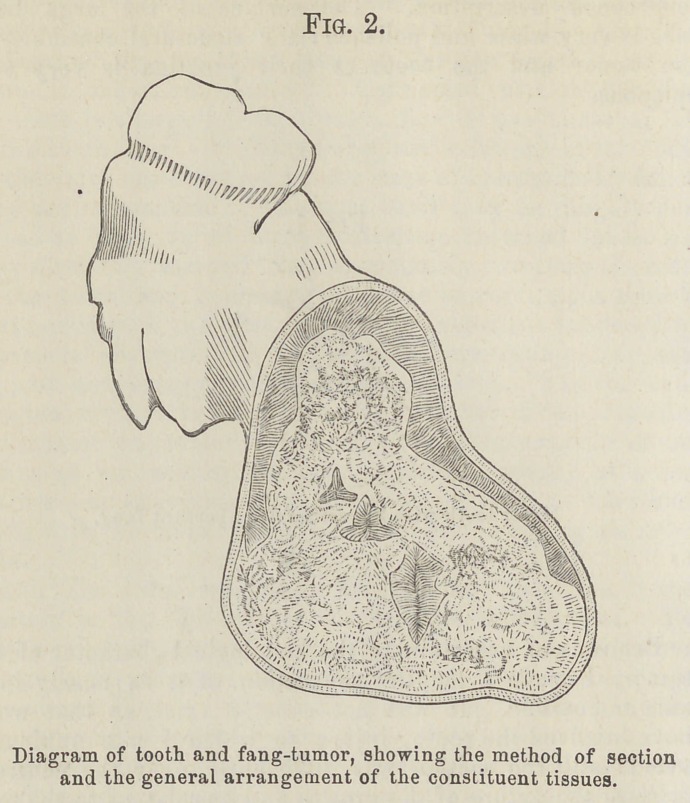# On the Structure of Two Forms of Tooth Tumor

**Published:** 1869-09

**Authors:** S J. A. Salter

**Affiliations:** F.R.S.


### Selections.

On the Structure of Two Forms of Tooth Tumor.—S J. A. Salter, M.B., F.R.S.—In December, 1867, M. P. Broca read a paper before the Academy of Sciences of Paris, under the title "Recherches sur un nouveau groupe de Tumeurs désigné sous le nom d'Odontomes," in which he described some of the hard excrescences which hypertrophy and abnormal growth of the tooth tissues produce. None of the abnormalities described by M Broca were new, though the arrangement of them under the one head—tooth-tumors—had not been before adopted by authors. Moreover, M. Broca's list was very incomplete, as no reference to either of the forms of tooth-tumor I am about to describe appears in his paper. Still, I think it must be conceded that to arrange all the tumors formed by the increased and perverted growth of the tissues of the teeth, under one head, is rational and expedient, and that the term "Odontome" is a convenient and legitimate expression.

The two Odontomes I am about to describe differ very materially in practical importance, while they are both of considerable physiological interest. Both are congenital. The one is extremely rare, but from its size is likely, when it occurs, to entail the necessity of serious surgical interference: the other is so minute and apparently so trivial, that the term 'tumor' might perhaps seem scarcely applicable to it; still, the expression may be fairly used; and the list of tooth-tumors would be incomplete without a description of this the smallest example:

*1.—A Tooth Tumor consisting of an Hypertrophied, Aberrant Fang.*

I believe that this heading best expresses the nature of the growth I am about to describe.

It is a specimen of disease of very great interest: it is extremely rare, and the only instances, which I believe to be similar, have been misunderstood.

The tooth with the tumor attached to it constitutes preparation 1022 of the Museum of the Royal College of Surgeons of England, and is believed to have been in the collection of John Hunter.

The Museum Committee of the College have very kindly allowed me to conduct these investigations.

As regards the rarity of this monstrous growth there are, I believe, three examples known in existence—neither more nor less—and all have, I venture to think, been misinterpreted. I feel that I ought to make this statement with great deference, considering the authorities who have described the specimens in question : at the same time I have no doubt about it in my own mind. The examples to which I have alluded are—

1st. One described by M. Forget.[1] It consisted of a large tumor, about the size of a bantam's egg, attached to the posterior surface of a lower molar tooth, adherent to the neck and a considerable portion of the fang. This specimen was taken from the mouth of a Frenchman, 40 years of age, who came to Paris to have the tumor removed on account of the annoyance it occasioned. The tumor occupied the left side of the lower jaw, expanding its sides, especially the outer, and disfiguring the face.

M. Maisonneuve, who attended the patient, determined to extract the tooth as a preliminary step to removing the tumor: the tooth, however, and the tumor came away together.

A section of the specimen through its entire length shows a complete continuity of tissue between the two, and the part in the illustration where the tumor and tooth are united is singularly like that seen in the specimen in the Museum of the College of Surgeons. The tumor is said by Forget to be composed wholly of osseous tissue.

2nd. The second example is recorded by Mr. Tomes in a paper read by him before the Odontological Society of Great Britain, April 6th, 1863.[2] The specimen was presented to the Odontological Society by Mr. Hare of Limerick. "The

---

1. 'Des Anomalies Dentaires, et de leur influence sur la Production des Maladies des Os Maxillaires,' par M. Forget, Paris, 1859. Obs. III, p. 27, pl. ii. figs. 1 and 2.

2 Description of a "Remarkable case of Exostosis," by J. Tomes, Esq., F.R.S., in 'Transactions of the Odontological Society of Great Britain,' vol. iii, p. 335. London, 1863.

tooth, a molar, was taken from the upper jaw of a countryman, 41 years old, who for some years previously had suffered severe pain in the jaw. The cheek was perforated by a canal through which matter constantly poured. After the removal of the tooth the pain in the jaw ceased, and the wound in the cheek healed." Connected with the fangs of the tooth is a large lobulated mass, four or five times as big as the tooth itself. "The number and relations of the roots of the tooth are obscured by the mass of cementum by which they are surrounded. The mass itself may be roughly described as built up of three coalescing flattened lobes, not very distinctly marked: one immediately investing the roots of the tooth, and composed of dense cementum; a second, continuous with the first, marked by abrasions produced by superficial absorption of the tissue, and presenting an appearance of less density than the preceding lobe. The third and terminal division is double the size of either of the preceding portions of the tumor." Though Mr. Tomes speaks of this tumor as an exostosis—it being composed wholly of crusta petrosa—no examination of its tissues appears to have been made with the microscope. The opinion is merely an inference.

3rd. The third specimen, which I now describe, is that existing in the Museum of the College of Surgeons. The only published reference to it with which I am acquainted occurs in Mr. Heath's admirable work on 'Diseases and Injuries of the Jaws.'[1] Mr. Heath, perceiving the similarity and the apparent identity of this tumor with those figured and described by Forget and Tomes, includes it in the same category, and describes it as a large exostosis. "In the Museum of the College of Surgeons is a specimen of large exostosis, due to hypertrophy of cementum."

This preparation consists of a rather small molar tooth from the posterior fang and neck of which passes off a large lobulated tumor, flattented from without inwards, more than twice the size of the tooth itself. The continuity of the tissue of the two is complete: the tumor is adherent to the tooth for its entire thickness from side to side. The form of

---

1 'Injuries and Diseases of the Jaw,' the Jacksonian Prize Essay of the Royal College of Surgeons of England, 1867, by Christopher Heath, Esq. F.R.C.S., London, 1868.

this adventitious growth and its relation to the tooth will be better understood by the accompanying figure than by any lengthened description. The surface of the large distal lobe is very white and polished: the structural continuity of the tumor and the tooth at their junction is very conspicuous.

Fig. 1.

Molar tooth with hypertrophied aberrant fang.

Desirous of ascertaining the histological character of this tumor, I made a lengthwise section of it as nearly in its axis as possible. It was not strictly axial, as that would have involved the tooth, injury to which I was anxious to avoid: still the section was sufficiently near the centre to disclose the nature of the growth and its relation to the tooth to which it was attached.

In grinding down the specimen, a small portion of the thin layer broke away from the narrow extremity: this does not, however, interfere with the demonstration of its structure, as I had already proved by repeated examinations with low magnifying powers, what were the histological elements constituting the section before it was sufficiently thin for permanent mounting.

The woodcut (Fig. 2) shows with perfect fidelity what is the structure of this tumor. The former is especially intended to display the relation of the parts of the growth to the tooth to which it is attached; and, though somewhat diagrammatic, is nevertheless strictly true.

A section of the tumor in the direction indicated in this figure (Fig. 2) shows the outer layer to be composed of a

Fig. 2.

Diagram of tooth and fang-tumor, showing the method of section and the general arrangement of the constituent tissues.

coat of crusta petrosa or cementum : it is even, compact, and of the usual character seen on tooth-fangs. Within this is a layer of true dentine: this does not constitute the entire circle of the section, but for about two-thirds of its circumference separates the external cemental layer from the *nucleus* of the growth, as it may well be styled. It is the two-thirds towards the attachment of the tumor to the tooth. For the remaining third there is no limitary band of dentine separating the nucleus from the crusta petrosa; but the line of demarcation between the two is perfectly distinct.

Upon examining the tissues of this section with high microscopic powers, the nature, the meaning, and the relation of these several histological elements are quite clear, as is their source of developmental production.

The outer layer is one of ordinary crusta petrosa, such as is seen on healthy tooth-fangs: it is laminated, non-vascular, and with the usual scattering of lacunæ, parallel to the axis of the laminæ.

The layer of the dentine is equally conspicuous and unmistakable; and, as is usual, the tubes have a general direction at right angles to the pulp-cavity and to the external surface of the growth of which they form part.

The nucleus of this odontome, its structure as displayed by high magnifying powers, and the inference as to its nature and source, constitute the most important points of interest in the specimen: they involve its meaning—the question what it really is.

1st. The *structure* of the nucleus. It is highly vascular, and the arrangement of the vessels is like that of the tooth-pulp; they branch and unite, and diverge in every conceivable direction; and their average diameter is about that which is seen in an uncalcified dentinal pulp. As regards the minute elements of structure, lacunæ largely prevail, and frequently occupy the whole field of the microscope: they are, however, somewhat peculiar, being large, without axial definition, and surrounded by crowds of canaliculi, looking like patches of moss. From this extreme form there is every conceivable variety of shape, passing by degrees to distinct and unmistakable dentinal tubes. Again, in other parts of the nucleus, isolated patches of true dentine are to be found, and some of these remote from the dentinal band and close to the crusta petrosa which bounds the bulbous end of the tumor. Moreover, in many parts there are masses of those calcification globules characteristic of dentine. In fact, the nucleus is composed of a confused mass of bone-structure and dentine-structure, arranged around and separating an elaborate vascular network of the same character as that of a dentinal pulp.

2nd. As to what may be inferred regarding the nature and source of this structure. It must be observed that the nucleus is *embraced within* a belt of true and unmistakable dentine:—that for two-thirds of its limit it is thus separated from true tooth-bone: that it is essentially different from the crusta petrosa hard by. It must be remembered further that whereas bone lacunæ may be found in a calcified dentinal pulp, dentine is never found in an exostosis—is never produced by the periodontal membrane.

In a paper which I published in the 'Guy's Hospital Reports' (1855), "On the intrinsic calcification of the tooth pulp," I showed that the dentine pulp, when it had undergone calcific impregnation of its whole structure, would often yield a mixture of bone tissue and dentine; and I figured one specimen, that of a temporary molar long retained in the mouth, and whose pulp had become calcified, in which the axis of the tooth did present this mixture of dentine and bone. Now, it would be impossible to distinguish the calcified pulp of this tooth from portions of the nucleus of the tumor I am now describing if they were placed under microscopes side by side.

I have no hesitation in saying that this nucleus was produced by the intrinsic calcification of a large dentinal pulp, of the same size and form as the nucleus; that the belt of dentine was the primary and normal development of that pulp; and that this hypertrophied and abnormal pulp, ceasing to contract and prolong centripetally the dentinal tubes, underwent a confused bone and ivory genesis, retaining its then vascular condition. It is scarcely necessary to refute the idea of this being an exostosis. Dental exostosis is entirely external, superficial to the ivory of the tooth: it is an extraneous growth deposited outside the dentinal system and in no way affecting it. A section of a tooth fang, however, incrusted with exostosis, has its dentinal element unaltered. Exostosis is a secondary affection occurring in after life. This expanded cone of dentine necessarily involved an original development of the same form—as dentine grows from without inwards; while crusta petrosa forms from within outwards.

I have expressed my belief that both the specimens described by Forget and Tomes are of the same nature as this specimen and that they are not exostoses, as stated; and I have come to this conclusion for many reasons. Their similarity—there is a close resemblance between the specimen I have described and the other two instances; it is especially like M. Forget's. In the latter case, too, the growth sprouts from the neck of the tooth principally, where exostoses do not occur. Again, there is nothing to lead to the idea that these are exostoses; there are no intermediate forms, nothing between the incrustations and small nodular tumors, which really are exostoses, and these large calcified masses. If these were exostoses, as stated, we should expect to find smaller ones of the same nature and more frequently: but that is not so.

M. Forget states that the structure of his specimen was proved by the microscope to be exclusively osseous tissue. I can not, however, help feeling a doubt whether the whole area of the cut specimen was scrutinised with exhaustive care. The accidental failure to examine a small space just within the outer coat of the tumor would lead to a total misapprehension of its nature. In the specimen I examined I obtained sections from the nucleus which were osseous nearly altogether, or with such faint indications of the dentinal element as easily to elude the observation of a microscopist, not anticipating their presence. As regards Mr. Tomes's specimen his statement that it is an exostosis amounts to nothing, as he did not examine its tissues with the microscope. I very much wish that both Forget's specimen and the one described by Mr. Tomes could be very carefully examined histologically and in all their parts.

There is one point in reference to Forget's and Tomes's specimens of interest and of anatomical value: they both display hollows or cavities, and, in the latter, the bulbous extremity of the tumor was little more than a hollow calcified cyst. This is never seen in true exostosis; but it is quite consistent with the idea of an hypertrophied, expanded, tooth-fang, whose pulp had not undergone calcification; it would be the equivalent of the specimen I have described, in which the tooth had been removed before the nucleus had passed from a soft pulp to a calcified mass.

## Figures and Tables

**Fig. 1. f1:**
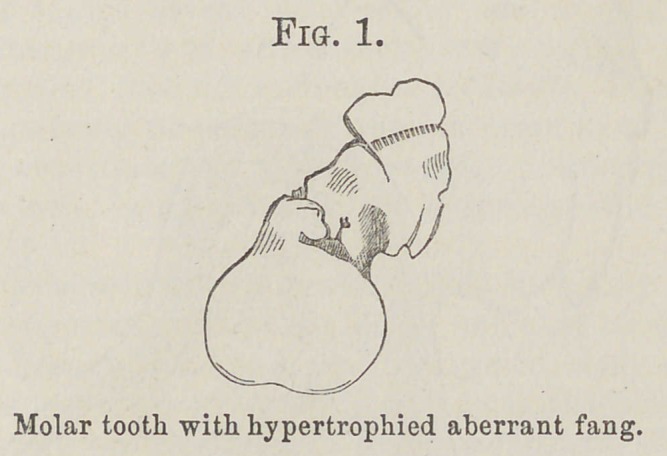


**Fig. 2. f2:**